# Baseline severe anaemia should not preclude use of zidovudine in antiretroviral-eligible patients in resource-limited settings

**DOI:** 10.1186/1758-2652-13-42

**Published:** 2010-11-03

**Authors:** Agnes N Kiragga, Barbara Castelnuovo, Damalie Nakanjako, Yukari C Manabe

**Affiliations:** 1 Infectious Diseases Institute, Makerere University, Kampala, Uganda; 2 Department of Medicine, Makerere University School of Medicine, Kampala, Uganda; 3 Division of Infectious Diseases, Department of Medicine, Johns Hopkins University School of Medicine, Baltimore, Maryland, USA

## Abstract

**Background:**

Stavudine is no longer recommended as part of first-line therapy for patients initiating antiretroviral therapy (ART) in Uganda. Most patients are currently initiated on zidovudine-containing regimens, which can induce anaemia. We investigated the risk factors for early severe anaemia in the first six months of ART initiation.

**Methods:**

We defined baseline (ART initiation) anaemia as haemoglobin (Hb) ≤9.5 g/dL, baseline severe anaemia as Hb ≤8 g/dL, and early severe anaemia as Hb ≤8 g/dL within six months of ART initiation. Risk factors for the development of early severe anaemia were analyzed using a multivariable logistic regression model.

**Results:**

In total, 5494 patients initiated ART, 821 (15%) had baseline anaemia, and 296 (5%) had baseline severe anaemia. Early severe anaemia occurred in 109 (4%) of 3105 patients who had at least one Hb measurement in the first six months on ART. Patients with baseline anaemia had a larger increase in Hb (median g/dL [IQR]) within the first six months compared with non-anaemic patients (2.9 [1.7, 4.6] vs. 0.7 [-0.2, 1.7], p < 0.0001). Having a new tuberculosis episode OR 3.69 (95% CI 1.64 - 8.32), MCV <80fL OR 1.60 (95% CI 1.01- 2.52) and baseline severe anaemia OR 5.27 (95% CI 3.00 - 9.26) were associated with early severe anaemia. Initiation on a zidovudine-based regimen was not associated with an increased risk of early severe anaemia.

**Conclusions:**

Among patients in an urban HIV clinic in Uganda, severe anaemia is modestly prevalent at ART initiation and improves with ART in the majority of patients. These data suggest that baseline severe anaemia should not be used as a criterion for avoiding the use of zidovudine in patients initiating ART in resource-limited settings.

## Background

Anaemia is a common condition in HIV-infected patients [1]. Before the introduction of antiretroviral therapy (ART), the prevalence of anaemia (defined as a haemoglobin level of less than 10 g/dL) ranges from 15% in asymptomatic patients to 50% in patients with a diagnosis of AIDS [1]. In addition, anaemia increases the risk for both morbidity and mortality in HIV cohorts [2-6]. Reports from the post-ART era in developed countries have shown substantial increases in mean haemoglobin (Hb) levels after ART initiation [7,8]. Although patients from resource-limited settings have lower baseline Hb levels compared with patients in studies conducted in resource-rich regions, the association between ART and the correction of pre-existing anaemia still holds [9-12].

Since 2008, stavudine (d4T) is no longer recommended as part of first-line therapy for patients initiating ART in Uganda due to short- and long-term toxicity [13]. At present, the recommended regimen is zidovudine (AZT) or tenofovir (TDF) plus lamivudine (3TC) plus a non-nucleoside reverse transcriptase inhibitor (NNRTI). However, due to the high cost and short supply of TDF, there is still significant continuing d4T use, and the majority of ART-eligible patients in Uganda are now initiated on AZT-containing regimens [14].

AZT has been reported as a cause of hematological disorders, especially anaemia [15,16], which most often occurs within 4 to 12 weeks of AZT initiation [9,12]. As a result of the introduction of World Health Organization (WHO) guidelines and the Uganda National Antiretroviral Treatment Guidelines for Adults, Adolescents and Children (which both recommend stopping AZT in patients whose Hb drops below 8 g/dL [17]), patients who have a Hb count of ≤8 g/dL) and advanced disease are initiated on TDF [18] rather than AZT, whenever possible.

The objective of our study was to analyze the risk factors for early severe anaemia, defined as Hb level ≤8 g/dL in the first six months after ART initiation, and, in particular, to examine if AZT could be safely prescribed to patients who initiate ART when they have baseline severe anaemia.

## Methods

Patients in this study were enrolled at the Infectious Diseases Institute, a centre of excellence in HIV care, treatment and research. The institute's clinic provided HIV-positive patients with free care and treatment, such as: counselling; clinical care; prophylaxis for opportunistic infections; laboratory testing, including CD4+ T cell count measurement; and antiretroviral treatment. ART was provided by the Multi-Country HIV/AIDS Program and the US President's Emergency Plan for AIDS Relief, and was prescribed according to WHO 2006 and Uganda Ministry of Health guidelines [13,17].

The first-line ART for adults and adolescents was d4T+3TC and nevirapine or efavirenz until March 2008 when AZT was recommended in lieu of d4T. Complete blood counts and CD4+ counts were performed every six months for all patients; HIV-RNA viral load measurements were not performed routinely, but could be requested by clinicians to confirm treatment failure. The Hb level was obtained using a Coulter counter (ACT V) on venipuncture samples.

Baseline Hb was defined as the most recent measurement taken within three months prior to ART initiation. Baseline anaemia was defined as Hb ≤9.5 g/dL consistent with the ACTG, Division of AIDS, National Institutes of Health grading [19], while baseline severe anaemia was defined as Hb ≤8 g/dL (the criteria for AZT discontinuation in Uganda, according to the Ministry of Health guidelines).

We analyzed the occurrence of early severe anaemia, Hb ≤8 g/dl within the first six months of initiation of ART. In our study, some patients did not have any other Hb measurement within six months of ART initiation. In order to minimize the bias from patients who died or were lost to follow up, we made every effort to characterize these patients with additional chart reviews to identify any symptoms of severe anaemia at the time of death or last study visit, in addition to other co-morbid conditions.

### Statistical analysis

We calculated the mean and standard deviation or median and interquartile range (IQR) of baseline characteristics and the change in Hb over the first six months after ART initiation. We used χ^2 ^tests to compare proportions and Student's t-test and Mann-Whitney test were used to compare continuous data. We used multivariable logistic regression to identify factors associated with development of early severe anaemia and included all patients who had an Hb test recorded within six months of commencement of ART. Patients with unresolved baseline severe anaemia were included in the multivariable analysis.

Baseline factors at AZT commencement included gender and age, body mass index (BMI), CD4 cell count, mean corpuscular volume (MCV), Hb, and ART regimen. Tuberculosis (TB) after ART initiation but before the development of anaemia was considered as a potential risk factor. Multivariable models were fitted using forward stepwise selection and variables with p < 0.10 or previously described risk factors being included in the model. Variables with multiple categories were included if overall tests for trend in heterogeneity were significant (p < 0.10).

In a separate analysis, we attempted to include all patients with missing Hb data within their first six months by imputing a "second" Hb measurement using chained equations (MICE), which involve creating sample missing values conditional on the distribution of remaining predictors in the multivariable model. We assumed that the Hb data in this group of patients was missing at random and carried out five rounds of multiple imputations; a combined dataset using Rubin's rule was then analyzed [20]. We compared the results from the imputation with those from the model without imputation. All the analyses were performed using STATA software, version 10.0 (STATA corporation, Texas, USA).

Ethical approval for the use of all routinely collected data for the care and management of HIV-positive patients at the Infectious Diseases Institute was obtained from the Makerere University Research and Ethics Committee and the Uganda National Council of Science and Technology.

## Results

### Baseline characteristics

We studied the data recorded for 5494 patients from our clinic who had baseline Hb measurements and were initiated on an AZT- or d4T-containing ART regimen between January 2004 and January 2009 (See additional file 1: description of patients at the Infectious Diseases Institute). The majority, 3264 (59.4%), were initiated on d4T-containing regimens. Of the 5494, 821 (15%) had baseline anaemia (Hb ≤9.5g/dL); 237 (28.9%) of the 821 anaemic patients were initiated on AZT. Among the 296 (5.4%) of the total 5494 patients who had baseline severe anaemia, 65 (22%) were started on an AZT-containing regimen, and 231 (78%) on d4T (p < 0.001) (See additional file 2: description of haemoglobin levels for patients on antiretroviral therapy).

Severely anaemic patients were more likely to be WHO Stage III-IV rather than I-II (252 (85%) vs. 44 (14.9%), p < 0.001), and were more likely to be female (208 (70%) vs. 88 (29.7%), p < 0.001). No blood transfusions were given to the patients with severe baseline anaemia. In our settings, only severely symptomatic anaemic patients are given blood transfusions, and they are not initiated on ART. Of the 296 patients with baseline severe anaemia, 65 (22%) were given haematinics (folic acid 5 mg and/or ferrous sulphate 200 mg) within three months of ART initiation; 52 were given both folic acid and ferrous sulphate; 12 were given only ferrous sulphate; and one patient was only given folic acid. Similar proportions of patients given haematinics were observed in the AZT and d4T group of patients: 13 of 65 (20%) versus 52 of 231 (22%), p = 0.665. Forty-three (66%) of those who were given haematinics had a low MCV (<80fl).

### Changes in haemoglobin after ART

The baseline characteristics of the 3105 patients who had a second Hb measurement within six months after ART initiation are listed in Table 1. Patients with baseline anaemia had a larger increase in Hb within the first six months compared with those who were non-anaemic (median change g/dL [IQR]; 2.9 [1.7- 4.6] vs. 0.7 [-0.2- 1.7], p < 0.0001). When we compared the median g/dL [IQR] changes in Hb for patients with baseline anaemia initiated on d4T and those on AZT, patients on d4T had a larger increase in Hb: 3.1 [1.8- 4.9] versus 2.5 [1.4- 5.0], p < 0.012). Median g/dL [IQR] changes within six months of ART initiation in patients with baseline Hb ≥11 g/dl was 0.3 [0.6- 1.2]; 8-11 g/dL was 1.5 [0.5- 2.5], and <8 g/dL was 3.55 [1.7- 5.3], p < 0.024.

**Table 1:** Baseline characteristics of patients with second haemoglobin measurement in first six months of ART initiation

Characteristic	All patients (n = 3105)	Patients with early severe anaemia (n = 109)
Gender		
Female	2065 (67%)	80 (73%)
Male	1040 (23%)	29 (26%)

Age (yrs) median (IQR)	36 (31,42)	37 (30,41)

BMI (kg/m^2^) median		
(IQR)	20 (18,23)	18 (17,22)

WHO stage		
I-II	937 (30%)	25 (23%)
III- IV	2168 (70%)	84 (76%)

CD4 T cell count,		
Median (IQR)	101 (33, 173)	65 (21,144)
200+	426 (14%)	12 (11%)
50-199	1589 (51%)	47 (43%)
<50	942 (30%)	44 (40%)
Missing	148 (5%)	6 (6%)

HB g/dL		
≥8	2959	79
<8	146	30

MCV, median (IQR)	84 (79,90)	79 (74,86)
≥80fL	2225	54
<80fL	880	55

TB		
No	3035 (98%)	93 (85%)
Yes	70 (2%)	16 (15%)

Regimen		
d4T-containing	1965	65
AZT-containing	1140	44

BMI = body mass index, HB = hemoglobin, MCV = mean corpuscular volume, IQR = interquartile range, TB = tuberculosis, ART = antiretroviral therapy, d4T = stavudine, AZT = zidovudine, OR = odds ratio

**Figure 1:**
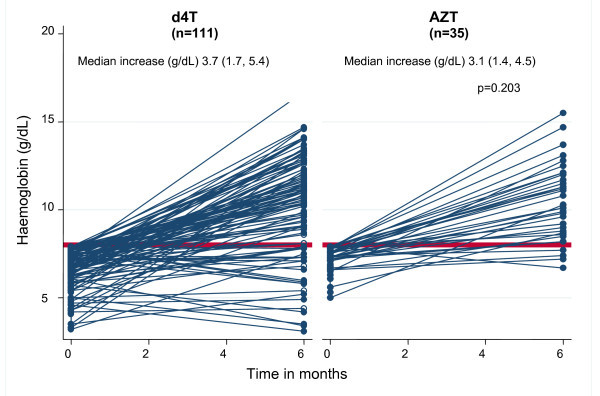
**Haemoglobin changes after ART initiation in patients with baseline severe anemia**. Change in Hb in first six months after ART initiation among patients initiated on d4t and with baseline severe anaemia (Hb ≤8 g/dL) and a second Hb measurement within six months of ART initiation.

Among patients with baseline severe anaemia, the 65% of those who were started on either regimen (AZT- and d4T-containing regimens) had increases in Hb to >9.5 g/dL. Figure 1 shows the change in Hb that occurs as a result of ART initiation by baseline regimen. When we compared the median Hb increases in patients with baseline severe anaemia who were initiated on d4T versus AZT, there were similar increases in Hb: (median g/dL [IQR]: 3.7 [1.7- 5.4] vs. 3.1 [1.4- 4.5], p = 0.203).

In the study, 3105 had second Hb measurements within six months on ART and of these, 109 (3.5%) had early severe anaemia (Hb ≤8 g/dl). Thirty (27.5%) of the patients with early severe anaemia had baseline severe anaemia and these had unresolved baseline severe anaemia. Only four of these patients with unresolved baseline anaemia had been initiated on AZT.

### Predictors of early severe anaemia

The univariate analysis showed that baseline severe anaemia Hb (≤8 g/dL), low MCV (<80fL), and having had a new TB episode after ART initiation were independently associated with increased risk of early severe anaemia (Table 2). The multivariable analysis showed that TB OR 3.69 (95% CI 1.64 - 8.32), MCV <80fL OR 1.60 (95% CI 1.01 - 2.52) and baseline severe anaemia OR 5.27 (95% CI 3.00 - 9.26) were significantly associated with early severe anaemia. Including AZT in the initial ART regimen was not associated with an increased risk of early severe anaemia compared with initiation of a d4T-based regimen OR1.33 (95% CI 0.85 - 2.07), when adjusted for age, gender, BMI, WHO stage, baseline CD4 T cell count, baseline Hb, MCV, and incident TB.

**Table 2:** Predictors of early severe anaemia in the first six months of ART initiation

Characteristic	Univariate OR (95% CI)	P-value	Adjusted OR (95% CI)	P-value
Gender				
Male	0.71(0.46-1.09)	0.122		

Age (yrs) median (IQR)	0.97(0.87-1.09)	0.662		

BMI (kg/m^2^) median (IQR)	0.89(0.84-0.96)	0.001	0.94(0.88-1.01)	**0.054**

WHO stage				
I-II	1.00		1.00	
III-IV	1.47(0.93-2.31)	0.095	1.23(0.72-2.09)	0.444

CD4 T cell count,				
Median (IQR)		0.057		0.606^a^
200+	1.00		1.00	
50-199	1.05(0.55-2.00)	0.878	0.84(0.41-1.72)	0.631
<50	1.69(0.88-3.23)	0.113	1.11(0.53-2.32)	0.782

HB g/dL,				
>8	1.00		1.00	
≤8	9.43(5.95-14.93)	<0.0001	5.27(3.00-9.26)	**<0.0001**

MCV, median (IQR)				
≥80	1.00		1.00	
<80	2.68(1.82-3.93)	<0.0001	1.60 (1.01-2.52)	**0.045**

TB episode				
No	1.00		1.00	**0.002**
Yes	8.96 (4.46-18.02)	<0.0001	3.69(1.64-8.32)	

Regimen				
d4T	1.00		1.00	0.209
AZT	1.17(0.79-1.73)	0.421	1.33 (0.85-2.07)	

**Note**: a - p value from overall test for trend in heterogeneity BMI = body mass index, HB = hemoglobin, MCV = mean corpuscular volume, IQR = interquartile range, TB = tuberculosis, ART = antiretroviral therapy, d4T = stavudine, AZT = zidovudine, OR = odds ratio

In a multivariable analysis stratified by gender, we found that a baseline severe anaemia OR 5.79 (95% CI 1.67 - 20.00), p = 0.006, and incident TB OR 5.68 (95% CI 1.89 - 17.14), p = 0.002, were associated with early severe anaemia in the male patients. In the female patients, the multivariable analysis showed again that incident TB OR 5.52 (95% CI 2.41-12.67), p < 0.0001, and baseline severe anaemia OR 5.51 (95% CI 2.86-10.60), p < 0.0001, were significantly associated with early severe anaemia. In addition, advanced WHO clinical Stage III-IV OR 2.08 (95% CI 1.01 - 3.97), p = 0.046, was also associated with severe anaemia. In this analysis, AZT was similarly not associated with early severe anaemia in both men and women.

### Outcomes of patients with baseline anaemia

Among patients with baseline anaemia who had second Hb tests, 232 (96%) of the 241 patients started on a d4T-containing regimen had an increase in Hb and nine (4%) had a decrease. Among the 92 patients started on an AZT-containing regimen who had baseline anaemia and at least one post-ART initiation Hb test, 85 (92.4%) had an increase in Hb and five (5.4%) had a decrease. When we consider only the subset of these patients who had severe baseline anaemia, of 111 patients started on d4T, 99 (89.2%) had an increase in Hb and 12 (10.8%) had a decrease. Of 35 started on AZT, 34 (97%) had an increase and only one (3%) had a decrease.

**Figure 2:**
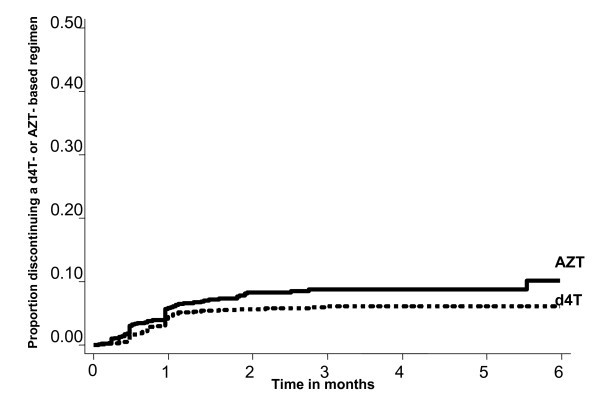
**Proportion discontinuing a d4T- or AZT- based regimen with six months of ART initiation**. Patients who were initiated on an AZT-containing regimen (n = 2230) are denoted with a dotted line; those initially on d4T-containing regimen (n = 3264) are denoted with a dashed line.

In the first six months, changes were made to the regimens of 134 patients; the switch rate per 100 person years at risk was 3.5 (95% CI 2.9 - 4.1). Overall, the rate per 100 person years at risk of regimen switching across baseline regimen (d4T vs. AZT) was not different (3.32 (95% CI 2.65 - 4.17) vs. 3.69 (95% CI 2.85 - 4.77), p = 0.749 (Figure 2). Switching due to toxicities was the main reason for treatment change in the first six months after initiation, occurring in 82 (61.2%) of 134; only one patient with baseline severe anaemia who began on an AZT-containing regimen had a regimen switch because of persistent or worsened anaemia.

### Characteristics of patients who did not have at least one Hb measurement

The 3105 patients had second Hb measurements within the first six months of ART initiation. Because exclusion of patients with only one Hb measurement could have excluded the patients with life-threatening anaemia and/or death after AZT initiation, we reviewed records for the 2389 patients who had only one Hb measurement at baseline and no additional Hb measurement during their first six months on ART. Of these, 2054 (86%) were still in active care at six months, and 335 (14%) were inactivated in the first six months of ART. Of the 335 patients, 154 (46%) had died, 160 (48%) had been transferred to other care providers, and 21 (6%) were lost to therapy; their baseline Hb was (median [IQR]) 11.4 g/dL (10.2, 12.6). Sixty-five (19.4%) had a baseline Hb <9.5 g/dL while 27 (6.6%) had baseline severe anaemia; 19 died and 17 were initiated on d4T. The median (IQR) Hb at start of ART for the patients who died was 10.6 (9.1-11.8). Anaemia clearly contributed to death in 11 of the 154 patients who died within six months of ART initiation, and five of these were initiated on d4T.

When we compared the baseline characteristics of patients who had only one Hb measurement within six months with those without second Hb tests in the six months after ART, the latter had lower median CD4 cells/mm^3 ^counts (101 versus 94, p = 0.008), more had started on AZT (46% versus 37%, p < 0.001), and they were less likely to be female (63% versus 67%, p = 0.006). In a multivariable analysis using the combined data set from the MICE procedure, which included all patients in the model after the imputation of a second Hb within six months for those patients without, we found that AZT was still not predictive of early severe anaemia; OR 1.43 (95% CI 0.92 - 2.21).

## Discussion

In our study, we found baseline anaemia in 15% of the patients who had Hb measurements within three months of ART initiation; one-third of the anaemic patients (5% overall) had severe baseline anaemia. Our results are similar to those seen in other sub-Saharan African centres that have initiated patients on ART, where the proportion of patients with baseline anaemia ranged from 12% to 18.2% [9], and the proportion with severe baseline anaemia ranged from 2% to 10% [21]. Results from the Development of Antiretroviral Therapy in Africa study showed that patients with BMI <18 kg/m^2 ^had a higher risk of developing Grade 4 (Hb <6.5 g/dL) anaemia [9]. A study in India showed that TB was also significantly associated with anaemia [22].

In our study, patients with baseline anaemia were more likely to present with TB after ART initiation suggesting that they may have had occult, unrecognized active disease or were sick at baseline and had not fully recovered and remained more susceptible. Among the 3105 patients with second Hb measurements, 70 had incident TB episodes before acquiring early severe anaemia; TB was associated with an increased risk for early severe anaemia, which suggested that anaemia was likely to be related to sub-clinical TB.

Although anaemia was prevalent in our population prior to the initiation of ART, ART led to an increase in Hb in the majority of our patients. This finding confirms data from studies in South Africa [12], Uganda and Zimbabwe [9], which report 0.28 g/dL and 0.6 g/dL median increases in Hb measured six months after ART initiation. In our study, the greatest Hb increases were realized in patients with the lowest baseline Hb levels, and only 3.5% developed early severe anaemia in the first six months of ART.

The most important risk factors for having early severe anaemia, in patients initiated on ART were a pre-existing diagnosis of TB, a low MCV, and baseline severe anaemia. In this resource-limited setting, AZT was not associated with an increased risk for early severe anaemia after highly active ART despite its known toxicity when used as a single agent in the pre-ART era. This finding has been previously described in the developed world [7,11].

In a sub-analysis of those patients who were initiated on AZT with a baseline severe anaemia, only one patient (of 65) had to be switched from AZT because of post-ART severe anaemia. This patient, with a baseline and nadir Hb of 6.8, was switched to d4T/3TC/EFV within two weeks of ART initiation and also developed a new TB episode, and was later transferred to another HIV treatment centre. In contrast, 12 of the 111 patients initiated on d4T developed worsening anaemia. The reasons that patients were initiated on AZT despite baseline severe anaemia are unknown and there may have been clinicians' reasons to start ART.

In a study conducted by Hoffman and colleagues in South Africa, none of the 11 subjects with Hb between 7.4 and 9.9 g/dL (who were not eligible to receive AZT, but were prescribed it) developed severe anaemia [12]. In the Treat Asia HIV Observational Database study in Asia, among patients started on an AZT-containing regimen, older age, lower BMI (<21 kg/m^2^), baseline anaemia (Hb <10 g/dL) and concurrent TB were associated with anaemia after ART, although this study did not examine AZT as a risk factor in all patients initiating ART [23].

The association of anaemia with concurrent TB has been shown in two studies: ours and that of Huffam *et al *[21]. These studies emphasize the importance of treating co-morbid diseases that can contribute to anaemia. Timely identification and treatment of TB should be accomplished through intensified case finding at the time of ART initiation [24]. Finally, both low MCV [8] and baseline anaemia [9,10,25] have been previously reported as risk factors for the development of severe anaemia in other studies that were conducted in the post-ART era and that corroborate our data.

In the univariate analysis, low BMI was also associated with an increased risk for post-ART anaemia, although this was not an independent risk factor in our multivariable analysis since it was confounded by other factors, including presence of TB and the very low baseline Hb. Other published reports showed BMI to be a risk factor for early severe anaemia [7], which may be a surrogate marker for patients who are more severely immunocompromised or with limited iron stores and protein to make Hb. Interestingly, a report from Thailand showed that switching to AZT from a d4T-based regimen after immune reconstitution is associated only rarely with AZT-induced anaemia [26]. Improvement of immunosuppression prior to switches to AZT-containing regimens, as evidenced by increased BMI and increased CD4 T cell counts, further decreased the risk of AZT-induced anaemia [27].

Although our study is based on data from a large prospective cohort that has been followed continuously since the beginning of ART rollout in Uganda, there are some limitations with respect to the generalizability of our conclusions. Patients who are initiating ART at present may have less immunosuppression (higher CD4 counts) than the cohort we analyzed. This difference may decrease the risk of AZT-induced anaemia [28].

In addition, the analysis only included patients who had both a baseline Hb measurement and at least one other measurement within six months of ART initiation. However, to minimize the bias from patients who died or were lost to follow up, we made every effort to characterize these patients with additional chart reviews and found that only 11 patients had symptoms of severe anaemia at the time of death in addition to other co-morbid conditions.

## Conclusions

In our cohort, the overwhelming majority of patients who initiated ART with any of the first-line regimens recommended in Uganda had improvements in Hb in the first six months. The development of early severe anaemia after ART initiation was most closely associated with baseline severe anaemia and incident TB infection and was not associated with the use of an AZT-containing regimen. Although we would conclude that AZT is relatively safe when compared to d4T, even in patients with severe baseline anaemia, if alternative regimens known to have even lower haematologic toxicity are available and affordable, they may be preferable.

Still, our study, taken together with other available data, shows that ART should not be withheld from patients with severe anaemia if regimens containing AZT are either the only ones available or are preferred for other reasons. Our data suggest that setting a lower limit Hb, specifically Hb ≤8 g/dL, as a determinant of whether AZT-containing regimens should be prescribed may not be warranted. Low BMI (<18 kg/m^2^) and low MCV (<80fL) may be more useful in predicting which patients are at highest risk for AZT-induced anaemia. Finally, intensified TB screening of anaemic patients is warranted, as well as vigilance for TB after ART initiation.

## Competing interests

The authors declare that they have no competing interests.

## Authors' contributions

AK performed the statistical analysis, and wrote the manuscript. BC and DN participated in its design and coordination, and helped draft the manuscript. YM conceived of the study, participated in its design and coordination, and helped draft the manuscript. All authors read and approved the final manuscript.

## Supplementary Material

Additional file 1 **Description of patients at the Infectious Diseases Institute**. Description of patients on antiretroviral therapy at the Infectious Diseases Institute from January 2004 to January 2009. Click here for file

Additional file 2 **Description of haemoglobin levels for patients on antiretroviral therapy**. Description of haemoglobin levels for patients on antiretroviral therapy at the Infectious Diseases Institute from January 2004 to January 2009. Click here for file
