# Supplementary material for: Baseline severe anaemia should not preclude use of zidovudine in antiretroviral-eligible patients in resource-limited settings
Source: J Int AIDS Soc. 2010 Nov 3;13:42. doi: 10.1186/1758-2652-13-42 (PMC2991285; doi:10.1186/1758-2652-13-42)
Supplement: Additional file 1 — Description of patients at the Infectious Diseases Institute. Description of patients on antiretroviral therapy at the Infectious Diseases Institute from January 2004 to January 2009. [file 1758-2652-13-42-S1.DOC]

**8031**

**5494**

2230 (40.6%) initiated on AZT

3264 (59.4%) initiated on d4T

821 (15%) with baseline anaemia

296 (5%) with baseline severe anaemia

2537- No baseline haemoglobin (Hb)*

**Baseline**

**6 months**

**post-ART**

*Baseline Hb is a value ascertained within three months of pre-ART initiation

2054 active at six months without Hb

154 dead

160 transferred

21 lost to therapy

**3105**

With Hb within first six months of ART
