# Supplementary material for: Baseline severe anaemia should not preclude use of zidovudine in antiretroviral-eligible patients in resource-limited settings
Source: J Int AIDS Soc. 2010 Nov 3;13:42. doi: 10.1186/1758-2652-13-42 (PMC2991285; doi:10.1186/1758-2652-13-42)
Supplement: Additional file 2 — Description of haemoglobin levels for patients on antiretroviral therapy. Description of haemoglobin levels for patients on antiretroviral therapy at the Infectious Diseases Institute from January 2004 to January 2009. [file 1758-2652-13-42-S2.DOC]

8031

5494

2537- No baseline Hemoglobin (Hb)*

AZT 2230

D4T 3264

Hb ≤9.5; 237

Hb >9.5; 1993

Hb ≤9.5; 584

Hb >9.5; 2680

**Baseline**

single Hb

112

2 Hb

125

single Hb

978

2 Hb

1015

single Hb

286

2 Hb

298

single Hb

1013

2 Hb

1667

**6 months**

**Post-ART**

*Baseline Hb is a value ascertained within 3 months of ART initiation

Hb ≤8; 65

Hb ≤8; 231

Hb ≤8; 11

Hb ≤8; 33

Hb ≤8; 36

Hb ≤8; 29
